# Scheduled dosage regimen by irreversible electroporation of loaded erythrocytes for cancer treatment

**DOI:** 10.1063/5.0174353

**Published:** 2023-10-16

**Authors:** Wencheng Peng, Yaqi Yue, Yuting Zhang, Hao Li, Cao Zhang, Peiyuan Wang, Yanbing Cao, Xiaolong Liu, Shoulong Dong, Ming Wu, Chenguo Yao

**Affiliations:** 1 State Key Laboratory of Power Transmission Equipment and System Security and New Technology, Chongqing University, Chongqing 400044, People's Republic of China; 2 The United Innovation of Mengchao Hepatobiliary Technology Key Laboratory of Fujian Province, Mengchao Hepatobiliary Hospital of Fujian Medical University, Fuzhou 350025, People's Republic of China; 3 Mengchao Med-X Center, Fuzhou University, Fuzhou 350116, People's Republic of China

## Abstract

Precise control of cargo release is essential but still a great challenge for any drug delivery system. Irreversible electroporation (IRE), utilizing short high-voltage pulsed electric fields to destabilize the biological membrane, has been recently approved as a non-thermal technique for tumor ablation without destroying the integrity of adjacent collagenous structures. Due to the electro-permeating membrane ability, IRE might also have great potential to realize the controlled drug release in response to various input IRE parameters, which were tested in a red blood cell (RBC) model in this work. According to the mathematical simulation model of a round biconcave disc-like cell based on RBC shape and dielectric characteristics, the permeability and the pore density of the RBC membrane were found to quantitatively depend on the pulse parameters. To further provide solid experimental evidence, indocyanine green (ICG) and doxorubicin (DOX) were both loaded inside RBCs (RBC@DOX&ICG) and the drug release rates were found to be tailorable by microsecond pulsed electric field (*μ*sPEF). In addition, *μ*sPEF could effectively modulate the tumor stroma to augment therapy efficacy by increasing micro-vessel density and permeability, softening extracellular matrix, and alleviating tumor hypoxia. Benefiting from these advantages, this IRE-responsive RBC@DOX&ICG achieved a remarkably synergistic anti-cancer effect by the combination of *μ*sPEF and chemotherapy in the tumor-bearing mice model, with the survival time increasing above 90 days without tumor burden. Given that IRE is easily adaptable to different plasma membrane-based vehicles for delivering diverse drugs, this approach could offer a general applicability for cancer treatment.

## I. INTRODUCTION

Stimuli-sensitive drug delivery system (DDS) has attracted tremendous attention due to its ability to allow drug release through a controlled manner in response to endogenous or exogenous stimuli, which can exert site-specific therapeutic effect while minimizing the side effects.^1^ Extensive efforts have been devoted to explore the ideal DDS capable of precise, timely, and targeted delivery of therapeutic agents to the lesion site without adverse effects on normal tissues in the body. Among them, erythrocytes have shown remarkable potential in drug delivery due to their inherent biocompatibility, low immunogenicity, especially as autotransplant, easy accessibility (most abundant cells in blood), long circulation, high *in vivo* stability, and membrane flexibility.^2–4^ In addition to therapeutic drugs, their organelle-free volume can be simultaneously loaded with other agents to provide multiple functions.^5,6^ Theoretically, endogenous triggers are fundamentally preferred based on the pathological differences between normal and diseased tissues, like pH gradients, redox potential, hypoxia, and enzymes, due to the advantages of self-controllability without the assistance of physical devices that may be harmful to patients or be tedious to operate.^7^ However, this approach requires a sophisticated molecule design of biocompatible materials featured with structural rupture or chemical cleavage in response to the desired stimulus. In addition, the internal stimuli in diseased areas are often not certain and distinct compared with normal tissues. These obstacles make the controlled drug release extremely difficult under *in vivo* physiological conditions.^8^ To achieve remotely controlled behavior in a specific spatial and temporal way, DDS in response to exogenous triggers like temperature, magnetic field, ultrasound, light, and electric field (EF) has been developed.^9^ For instance, thermosensitive liposomes (ThermoDox) have been approved for breast (phase II) and hepatocellular cancer (phase III) treatment in clinical trials.^1,10^ Despite great potential, on-demand controlled output (drug release rate) selectively activated by physical input (external stimuli) is still a huge challenge, because of various hindrances related to insecurity, limited penetration, poor selectivity, neither direct nor usable interoperability, sophisticated or toxic chemical cofactors, etc.^11^

Irreversible electroporation (IRE),^12^ as a non-thermal interventional technique causing cell death while preserving the integrity of adjacent tissue structures like vessels, nerves, and bile ducts, has shown great potential for locoregional treatment of various tumors including liver cancer, squamous cell carcinoma, pancreatic ductal adenocarcinoma (PDAC), basal cell carcinoma, prostate cancer, and so on.^13–17^ The principle of IRE to ablate tumor tissue is to employ a series of high-voltage microsecond pulsed electric field (*μ*sPEF) to selectively create permanent membrane damage on cancer cells.^18,19^ Yet, owing to the security concerns (i.e*.,* muscle contractions and cardiac arrhythmia), the therapeutic efficiency of this innovative technique is still modest as tumor tissues cannot fully be exposed to a strong electric field.^20^ To reduce these risks, we developed an innovative therapeutic apparatus that uses bursts of high-frequency alternating polarity pulses (HF-IRE) to replace the traditional IRE. The first human trial of HF-IRE for treating prostate cancer confirmed that HF-IRE can effectively ablate the tumor without visible muscle contractions during the pulse delivery process.^21^ In addition to directly damaging the cell membrane, *μ*sPEF might offer a promising solution toward the *in situ* release from cell-based DDS as a switch to control the degree of membrane damage, given that many different types of electric field have been used to control cargo release with the advantages of minimal invasiveness, easy manipulation, low cost, and not any involvement of complex enhancers.^11,22^ Additionally, *μ*sPEF may also synergistically promote the outcomes of other therapeutic modalities through changing the structure and composition of the tumor microenvironment (TME). For instance, Zhao *et al.* found that IRE was able to downregulate some factors associated with the fibrotic stroma, including FAPα, hyaluronic acid, and LOX fibrotic, to potentiate immune checkpoint blockade effect in PDAC.^15^ In addition to immunotherapy, other therapeutic modalities such as photodynamic therapy (PDT), chemotherapy, and radiotherapy might also benefit from TME changes created by IRE.^23–26^ Hence, exploiting *μ*sPEF to directly trigger drug release from membrane-based DDS would augment cancer therapeutic effect from multiple aspects.

**FIG. 1.**
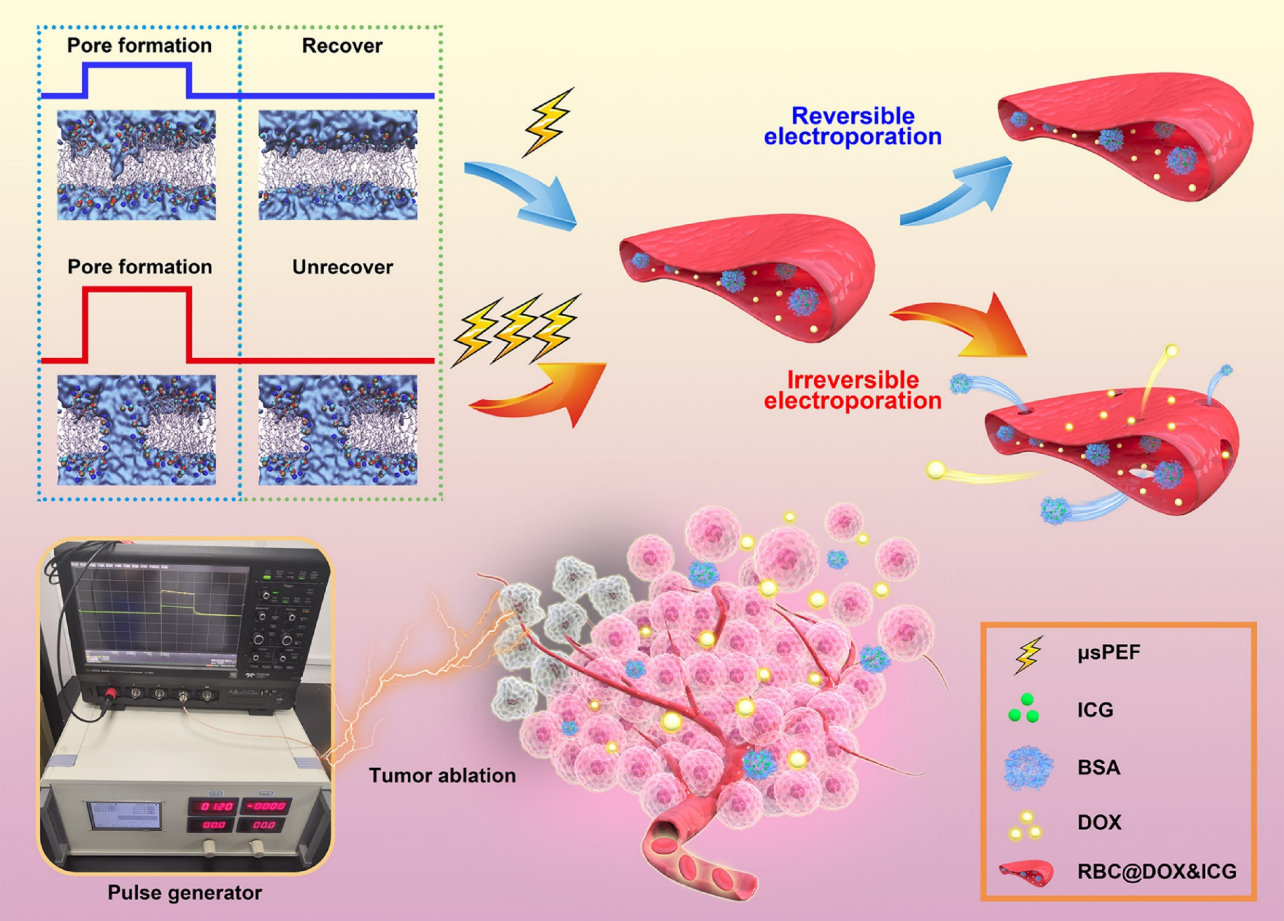
Schematic illustration of the *μ*sPEF controlled drug release using RBCs as delivery vehicles for cancer treatment.

As a proof of concept, we aimed to establish a scheduled dosage regimen by exploiting *μ*sPEF-controllable DDS for cancer therapy in this work (Fig. 1). For this purpose, red blood cells (RBCs) were first selected as a cell-based DDS model, because they can be easily loaded with diverse drugs with the merits of biocompatibility, low immunogenicity, easy accessibility, long circulating, etc.^27–29^ According to the 3D simulation by finite-element analysis based on RBC shape and dielectric characteristics, the permeability and pore density of plasma membrane were found to quantitatively depend on the pulse parameters. Furthermore, to validate this approach, we loaded indocyanine green (ICG, fluorescence imaging probe) and doxorubicin (DOX, chemotherapy drug) inside RBCs and then evaluated their drug release behavior and anticancer performance. The results certified that IRE could activate the RBC-based DDS to inhibit tumor growth without recurrence even up to 90 days, through IRE-mediated tumor ablation, controlled drug release, and TME modulation. Given that both IRE and cell transplantation are already in clinical use, this IRE-controllable DDS strategy would be realizable for individually optimized cancer treatment.

## II. RESULTS

### A. 3D simulation of the RBC characteristics under *μ*sPEF

**FIG. 2.**
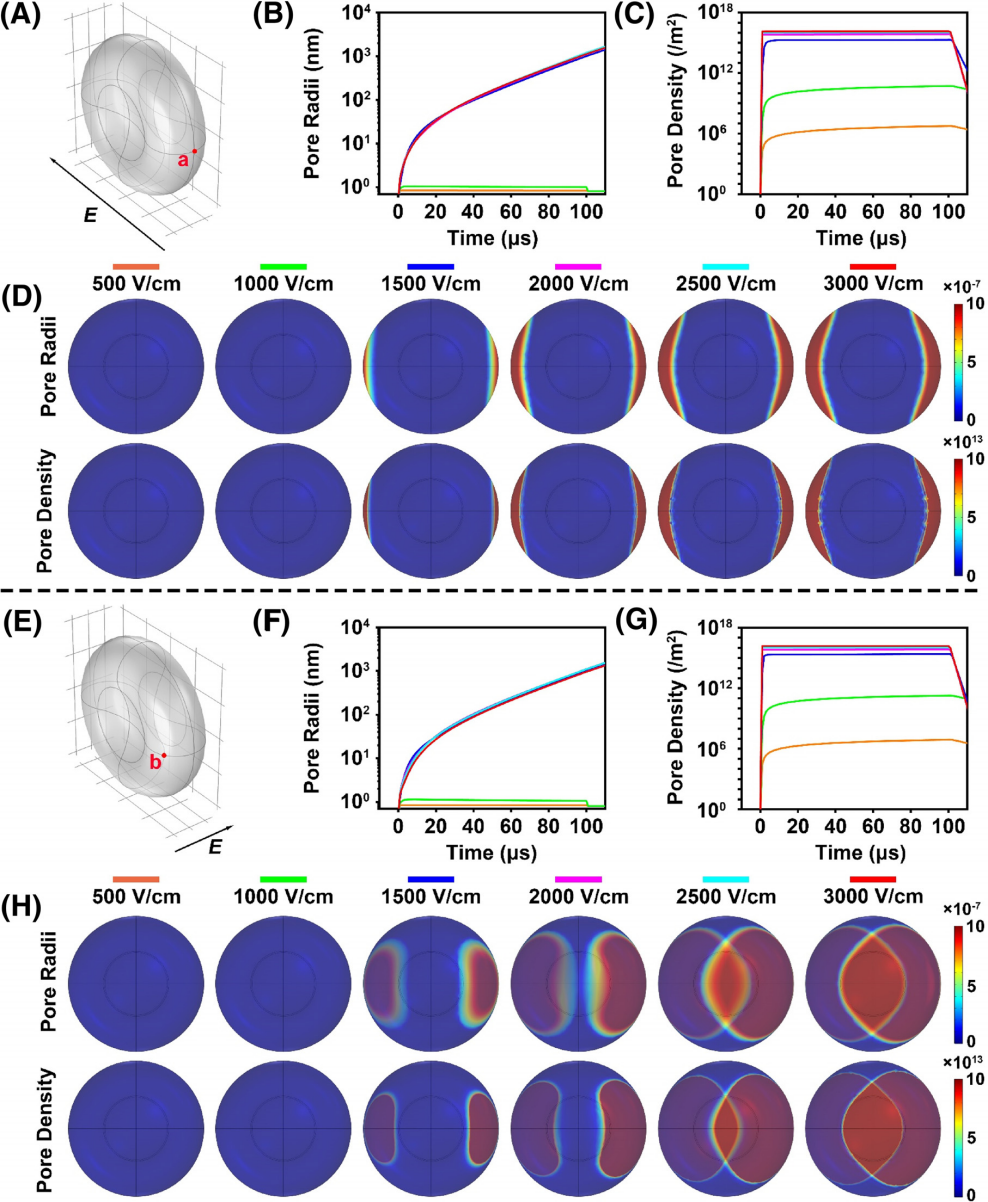
Simulation of a single RBC under *μ*sPEF. There were two situations in which the RBC was exposed at two distinct electric field directions perpendicular to each other. With the increase in the electric field strength, the electroporation region (red) became larger. Above the dotted line: (a) finite-element model of the RBC under microsecond pulsed electric field (direction: outside-to-in), with point *a* labeled in convex edge. (b) and ( c) The change in pore radii and pore density at point *a*, with varying intensity of *μ*sPEF. (d) The distribution of the electroporation region at 100 *μ*s after the pulse. The blue area is the surface of the RBC where point a and b are located. The red region shows the electroporation region. Below the dotted line: (e) finite-element model of the RBC under microsecond pulsed electric field (direction: left-to-right), with point *b* labeled in concave center. (f) and (g) The change of pore radii and pore density at point *b*. (h) The distribution of the electroporation region at 100 *μ*s after the pulse. The blue area is the surface of the RBC where point *a* and *b* are located. The red region shows the electroporation region.

According to our previous studies with some modifications,^30^ a three-dimensional finite-element model of the RBC exposed to microsecond pulsed electric fields was first constructed for exploring the membrane electroporation characteristics. The relationship between the pulse parameters and electroporation was deeply investigated to explain the on-demand controlled drug release by *μ*sPEF. We simplified a single RBC exposed to a pulsed electric field with strength *E* in a conductive medium. The theoretical data were calculated from COMSOL Multiphysics by the electricity and transient analysis application mode. To monitor the progression of pore radii and pore density on RBC membrane, two representative positions of *a* and *b*, respectively, located in convex and concave of RBC model were selected according to the two distinct electric field directions perpendicular to each other when the RBC was exposed, as shown in Figs. 2(a) and 2(e). With respect to position *a*, when the RBC was exposed to *μ*sPEF with duration of 100 *μ*s and various field strengths, the pore evolution was found to depend on the electric field intensity. As shown in Fig. 2(b), the pores expanded gradually with elevation of the electric field. Under the external electric field, more hydrophobic pores would form in the membrane at the beginning. Then, these hydrophobic pores would gradually become hydrophilic as the strength of external electric field increased to finally form stable electroporation. When electric field strength increased above 1500 V/cm, the pore densities and pore radius on the RBC membrane elevated along with the electric field strength [Figs. 2(c) and 2(d)]. Figure 2(d) presents the distribution of pore radii and pore density on the surface of the RBC after application of pulsed electric field. With the increase in the electric field strength, the electroporation region (red) became larger. In position *b*, the trend of pore evolution was similar to that of position *a*, also presenting a positive correlation between electroporation and electric field strength [Figs. 2(f) and 2(g)]. However, the electroporation region in Fig. 2(e) is much larger than Fig. 2(a), indicating that most electroporation occurred in the concave of RBC under the same pulse [Figs. 2(d) and 2(h)]. Collectively, these results provide a theoretical basis for the tailorable drug release in the following experimental study or even clinical trial in the future.

### B. Characterization of RBC@DOX&ICG

**FIG. 3.**
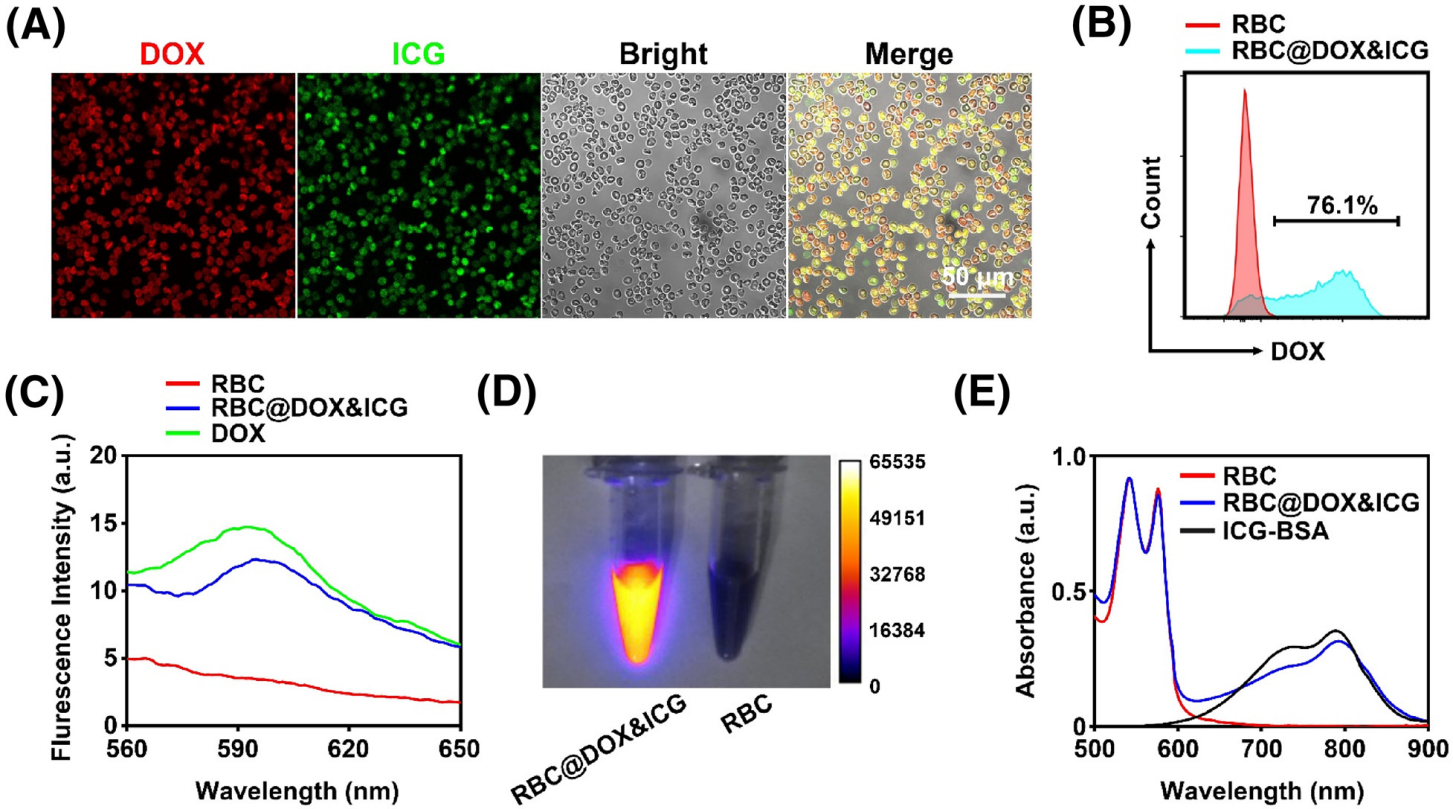
Characterization of RBC@DOX&ICG. After the “hypotonic dialysis/hyperosmotic recovery” treatment, DOX and ICG-BSA were loaded into RBCs. RBC@DOX&ICG could be visualized by the confocal fluorescence microscope and analyzed using flow cytometry, fluorescent spectrum, and absorption spectrum. (a) Confocal images of RBC@DOX&ICG. Red and green fluorescence suggested DOX and ICG were successfully loaded into RBCs. (b) Flow cytometry data proved that 76.1% of RBCs were loaded with DOX. (c) The fluorescent spectrum of native RBCs, free DOX, and RBC@DOX&ICG in the range of 560–650 nm. (d) The NIR-II fluorescence images of native RBCs and RBC@DOX&ICG merged with the bright field. (e) The absorption spectrum of native RBCs, ICG-BSA, and RBC@DOX&ICG in the range of 500–900 nm.

To prove the on-demand drug release controlled by *μ*sPEF, we adopted the “hypotonic dialysis” method to load DOX and ICG-BSA into RBCs.^28^ DOX and ICG-BSA nanocomplexes could easily enter the swollen RBCs due to the enhanced permeability under the condition of hypo-osmolality. Following resealing in hyperosmotic medium, the RBCs could lock the DOX and ICG-BSA inside. Therefore, the as-prepared RBC@DOX&ICG can be simultaneously used for chemotherapy and fluorescence imaging. Drug-loading in RBCs was visualized via the confocal fluorescence microscope [Figs. 3(a) and S4]. The colocalization of DOX and ICG-BSA indicated that they were effectively encapsulated inside the RBCs. Flow cytometry data further determined that 76.1% of RBCs were loaded with DOX [Fig. 3(b)]. The fluorescence spectra confirmed the RBC@DOX&ICG had the same emission peak as DOX at around 595 nm, while control RBCs had barely any florescence [Fig. 3(c)]. High-performance liquid chromatography calculated that about 1.63 × 10^−16 ^mol of DOX was loaded inside per RBC [Fig. S5(a)]. The fluorescence image suggested that RBCs loaded with ICG possessed NIR-II emission ability under 808 nm laser irradiation [Fig. 3(d)]. According to the absorption spectrum [Figs. 3(e) and S6], RBC@DOX&ICG and ICG-BSA had the same absorption and emission peak at about 795 nm. Then, from the standard curve quantified by ICG absorption spectrum [Fig. S5(b)], it was estimated that around 8.84 × 10^−17 ^mol of ICG was loaded inside per RBC. In addition, phosphatidylserine (PS) exposure of RBCs' carrier was detected through annexin V-FITC staining method, to confirm whether RBCs remained intact in the drug-loading process as PS exposure had been widely recognized to involve in recognition and clearance of impaired RBCs by RES system.^31^ From the flow cytometry data in Fig. S7, the PS exposure of native RBCs or RBCs carriers with aforementioned treatment was negligible (less than 5%) in comparison with RBCs ghost as positive control treated by pure water (72.2%). In order to further study the effect of the above-mentioned treatment on native RBCs, the dye DiI was used to label native and treated RBCs, and labeled RBCs were injected into the mice. At different time points, the blood was collected from the mouse tail and imaged to detect the fluorescence intensity. As shown in Fig. S8, there was no difference in fluorescence intensity between the two groups, which meant that the treatment did not damage the circulation time of RBCs. Meanwhile, compared with positive control of lysed RBCs, the supernatant of RBCs with “hypotonic dialysis/hyperosmotic recovery” treatment contains little hemoglobin (Fig. S9). Furthermore, we also studied the sensitivity of the loaded RBCs to mechanical, oxidative, and osmotic stress. RBC resistance to mechanical stress is one of the key physiological features that contribute to RBC longevity in the bloodstream. Figure S10 demonstrates that the morphology of RBC@DOX&ICG remained predominantly intact and stable even after a 48-hour storage period. As shown in Figs. S11(a) and S11(b), it is indicated that the mechanical fragility of encapsulated RBCs is only slightly reduced compared to naive RBCs with the incubation time less than 4 h. Significantly, RBC@DOX&ICG has favorable effects against oxidative damage [Fig. S11(c)], which might be due to the antioxidation role of encapsulated ICG or DOX. Figure S11(d) showed that the sensitivity of drug-loaded RBCs to osmotic stress was reduced after undergoing the hypertonic and hypotonic procedures. In addition, RBC agglutination assay showed that the performance of RBC@DOX&ICG and naive RBCs was almost similar. These results indicate that the encapsulation procedures merely disrupt the membrane structure of RBCs, which is important to maintain their biological activity like long systemic circulation after intravenous administration.

### C. Drug release manipulated by *μ*sPEF

According to the 3D simulation of the RBC, the drug release from RBC@DOX&ICG can be controlled by IRE. To verify this, *in vitro* drug release experiments were carried out. The pulses were generated by pulse generation equipment.^32,33^ This equipment contained three parts: a pulse generator, a pulse measuring device, and a cell/animal stimulation module, as shown in Fig. S12. The pulse generator was designed and assembled in our laboratory, which was intelligently controllable and parameter adjustable. The measuring device contained an oscilloscope, a high-voltage probe, and Pearson current probe. For *in vitro* experiments, the electroporation cuvette was used to stimulate the cell suspension.

**FIG. 4.**
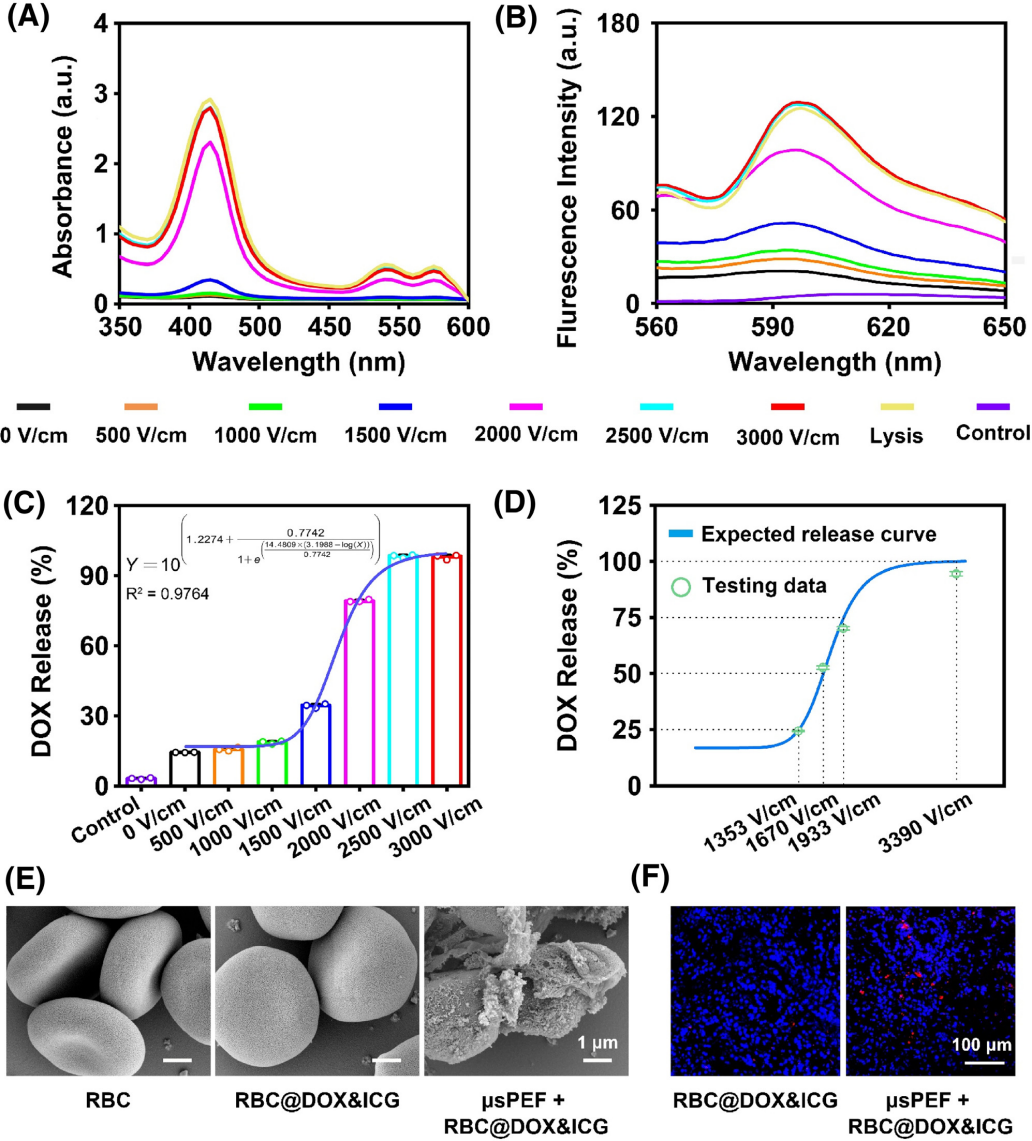
Controlled drug release by the electric field. (a) and (b) The absorption spectrum (350–600 nm) and fluorescent spectrum (560–650 nm) of the supernatant after RBC@DOX&ICG treated by the different intensity of *μ*sPEF. Unloaded RBCs exposed to *μ*sPEF at 2500 V/cm are set as the control group. (c) The release percentage of DOX under different intensity of *μ*sPEF (n = 3) through a standard curve of fluorescence intensity and the corresponding content of DOX. (d) Consistency analysis of the fitted curve and tested data of DOX release (n = 3). (e) SEM images of innate RBCs, RBC@DOX&ICG, and RBC@DOX&ICG treated with *μ*sPEF at 2500 V/cm. (f) The CLSM images of frozen sections of tumor tissues inserted with needle electrode to present *μ*sPEF at 2500 V/cm to monitor DOX release. After being encapsulated in RBCs, the fluorescence of DOX will be significantly quenched due to the aggregation-caused quenching (ACQ) effect, but after release triggered from RBCs by electric field, the DOX fluorescence will recover, as verified in Fig. S15. Thus, the fluorescence signal (red) in tumor sections could be used to monitor drug release.

After drug-loading, the *μ*sPEF was applied to the RBC@DOX&ICG suspension in a 2 mm-gap cuvette. Then the centrifugated supernatant was collected to determine the drug release from the RBCs carrier through the absorption spectrum and the fluorescence spectrum. Figures 4(a) and 4(b) showed that the stimulated RBC@DOX&ICG immediately released the hemoglobin (absorption peak at 415 nm) and the DOX (fluorescence emission peak at 595 nm) in response to *μ*sPEF, which had a positive correlation with the electric field strength. The release percentage of DOX under different electric field strength was shown in Fig. 4(c). The control group of unloaded RBCs, which are exposed to *μ*sPEF at the highest field strength, was used to prove that hemoglobin had hardly any autofluorescence to interfere with DOX fluorescence. At low electric fields of 500 and 1000 V/cm, there were only slightly enhanced drug release, about 15.7% and 18.7% compared with the control sample with 14.4% of DOX release. When the pulsed intensity reached 1500 V/cm, 34.3% of DOX was released. Most significantly, when the intensity was up to 2500 V/cm, almost all DOX (98.2%) was released, implying that 2500 V/cm was enough to completely destroy RBCs. On account of drawing a general rule of drug release trigged by *μ*sPEF, the logistic regression was used to describe the correlation of pulsed electric field intensity and drug release amount. The fitted curve was as follows:

$$Y=10^{\left(1.2274+\frac{0.7742}{1+e^{\left(\frac{14.4809\times (3.1988-\text{log}\;(X))}{0.7742}\right)}}\right)}.$$ (1)

To verify the feasibility of the release formula in turn, theoretically pulsed intensities were calculated according to the different release percentages scheduled at 25%, 50%, 75%, and 100%. Then, we determined the actual drug releases under these deduced pulsed intensities (1353, 1670, 1933, and 3390 V/cm). The strong consistency between the tested and expected data in Fig. 4(d) indicated that the drug release could be customized by adjusting the strength of pulsed electric field.

The photograph of the treated supernatant in Fig. S13 also supported the controlled drug release behavior in response to the pulsed electric field. The *in vitro* electric field-sensitive drug release can be explained by numerical simulation. As shown in Fig. 1, the simulation explained that minimal drug release was due to the limited pore radius and density at a lower electric field below 1000 V/cm. In addition, from the simulation and experimental results, 2500 V/cm seemed to be an optimal parameter that fully destroyed RBCs. In addition, the temperature of the RBCs suspension after pulse treatment showed that 2500 V/cm did not cause a dramatic temperature rise (Fig. S14), excluding the possibility of heat damage. Thus, we chose 2500 V/cm as the parameter to trigger DOX release in the following experiments. To study the morphology changes of RBCs before and after the drug-loading and *μ*sPEF treatment, the scanning electron microscope (SEM) was employed [Fig. 4(e)]. Native RBCs displayed normal biconcave shape, which still maintained a similar surface after loading with DOX and ICG, while treated RBCs had rough surface, and some RBCs became broken [Fig. 4(e)]. Additionally, *in vivo* drug release experiment was conducted. After intravenous injection of RBC@DOX&ICG into the tumor-bearing mice, an electric field of 2500 V/cm was applied. After 5 min, the tumors were collected for frozen sections to analyze DOX fluorescence (red). Figure 4(f) demonstrated that *μ*sPEF could manipulate DOX drug in tumor tissue. Figure S15 indicates that DOX and ICG are released from RBCs upon treatment with *μ*sPEF, resulting in fluorescence recovery. These results demonstrated that electroporation could be effectively induced on the RBC membranes by the external *μ*sPEF to realize the on-demand drug release.

### D. *In vitro* anti-cancer effect

**FIG. 5.**
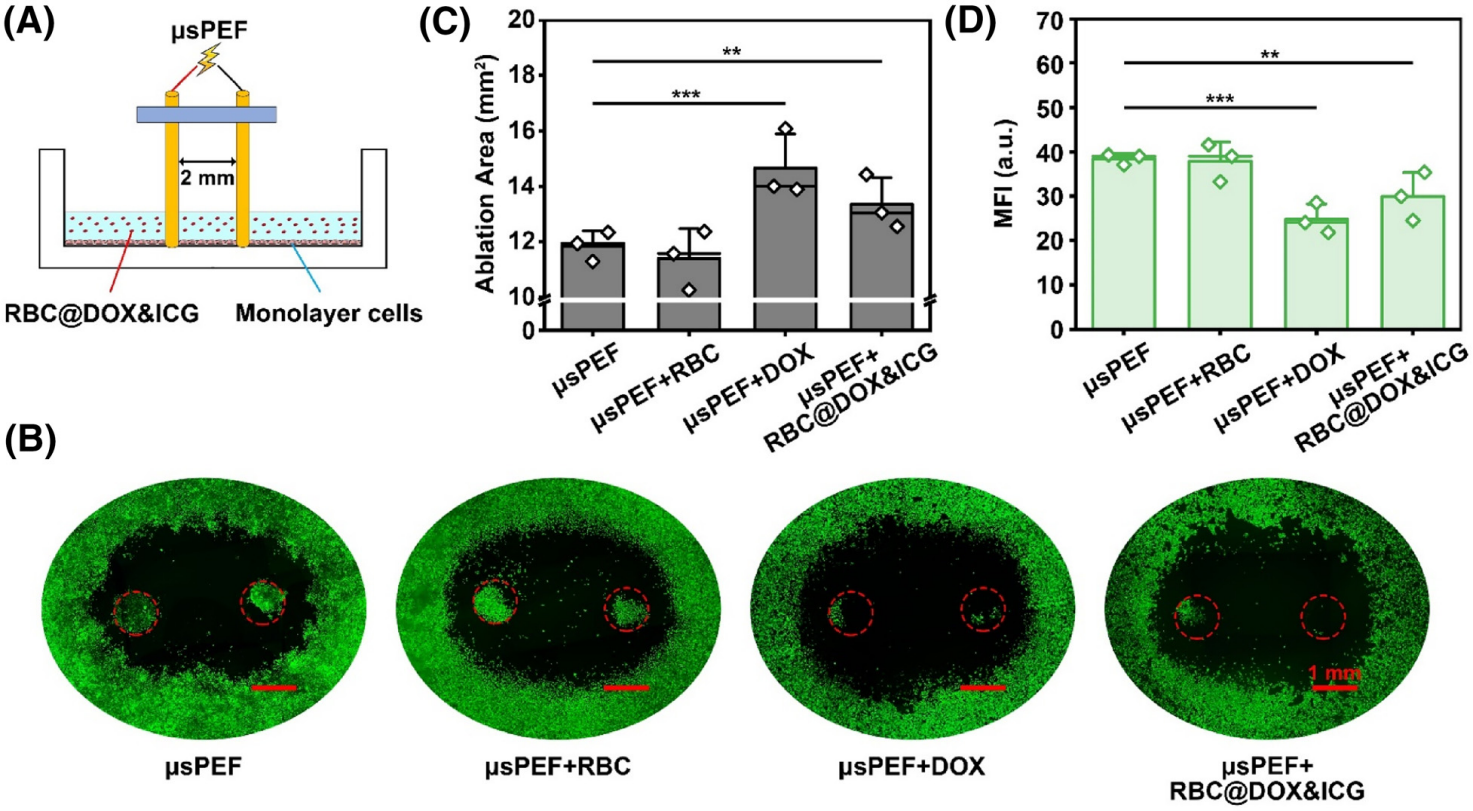
*In vitro* anticancer effect. To study the synergetic effect of *μ*sPEF at 2500 V/cm and RBC@DOX&ICG, monolayer cells were used to estimate ablation area. According to the results, μsPEF could effectively manipulate drug release and ablate cancer cells. (a) Schematic illustration of *μ*sPEF combining RBC@DOX&ICG to stimulate the monolayer cells. (b) Florescence image of Hepa 1–6 cells after 24 h of treatment with *μ*sPEF, *μ*sPEF+RBC, *μ*sPEF+DOX, or *μ*sPEF+RBC@DOX&ICG. (c) and (d) Ablation area and MFI calculated from (b) (^*^p < 0.05, ^**^p < 0.01, ^***^p < 0.001; n = 3).

The IRE-responsive DDS for cancer cell inhibition was demonstrated in monolayer cultured Hepa 1–6 cells using the needle electrode as depicted in Fig. 5(a). After incubation with unloaded RBCs, free DOX, or RBC@DOX&ICG, the *μ*sPEF (2500 V/cm) was introduced to ablate cancer cells and regulate DOX release from RBCs, while the cells only treated with *μ*sPEF at the same parameters were used as the control. The green areas were living cells stained with calcein AM and the black areas represented the ablation area of the *μ*sPEF after 24 h of treatment. According to Fig. 5(b), there was no significant difference in the ablation area between *μ*sPEF + RBC and *μ*sPEF alone, but the combination of *μ*sPEF with free DOX or RBC@DOX&ICG can enlarge the ablation area. Figure 5(c) displayed that *μ*sPEF could ablate monolayer tumor cells effectively, with a distinct ablation area of about 11.86 ± 0.53 mm^2^. The areas of *μ*sPEF + DOX increased to about 14.66 ± 1.24 mm^2^. In comparison, the combination therapy of *μ*sPEF and RBC@DOX&ICG resulted in ablation area of about 13.34± 0.97 mm^2^. The nearly identical levels of cytotoxicity observed between *μ*sPEF + DOX and *μ*sPEF + RBC@DOX&ICG can be attributed to the rapid release of most of the DOX from the drug-loaded red blood cells (RBC@ICG&DOX) following *μ*sPEF treatment. Therefore, there is no significant difference in cellular uptake between the RBC@ICG&DOX formulation and an equivalent concentration of free DOX in the presence of *μ*sPEF. Consistently, the mean fluorescence intensity (MFI) of the living cells dramatically declined after the combination of *μ*sPEF and RBC@DOX&ICG [Fig. 5(d)]. Additionally, the groups of untreated control, unloaded RBCs, RBC@DOX&ICG alone, and DOX alone were studied. As shown in Figs. S16 and S17, DOX alone can inhibit the proliferation of tumor cells and normal cells, while DOX release from RBC@DOX&ICG can hardly occur in the absence of *μ*sPEF. According to these results, *μ*sPEF could effectively manipulate drug release and ablate cancer cells.

### E. *In vivo* distribution and biosafety assay

ICG, as a NIR I/II fluorescent dye, has been successfully applied for image-guided surgery in clinic. With the guidance of NIR fluorescence, the location and boundary of tumors will be identified, which is helpful to place electrodes and determine pulse parameters for IRE ablation. Due to the prolonged circulation half-lives and the ability of immune escape, RBCs are excellent carriers for drug delivery. In this paper, RBCs were used as ICG carriers to improve tumor targeting and prolong tumor retention for fluorescent navigation.

**FIG. 6.**
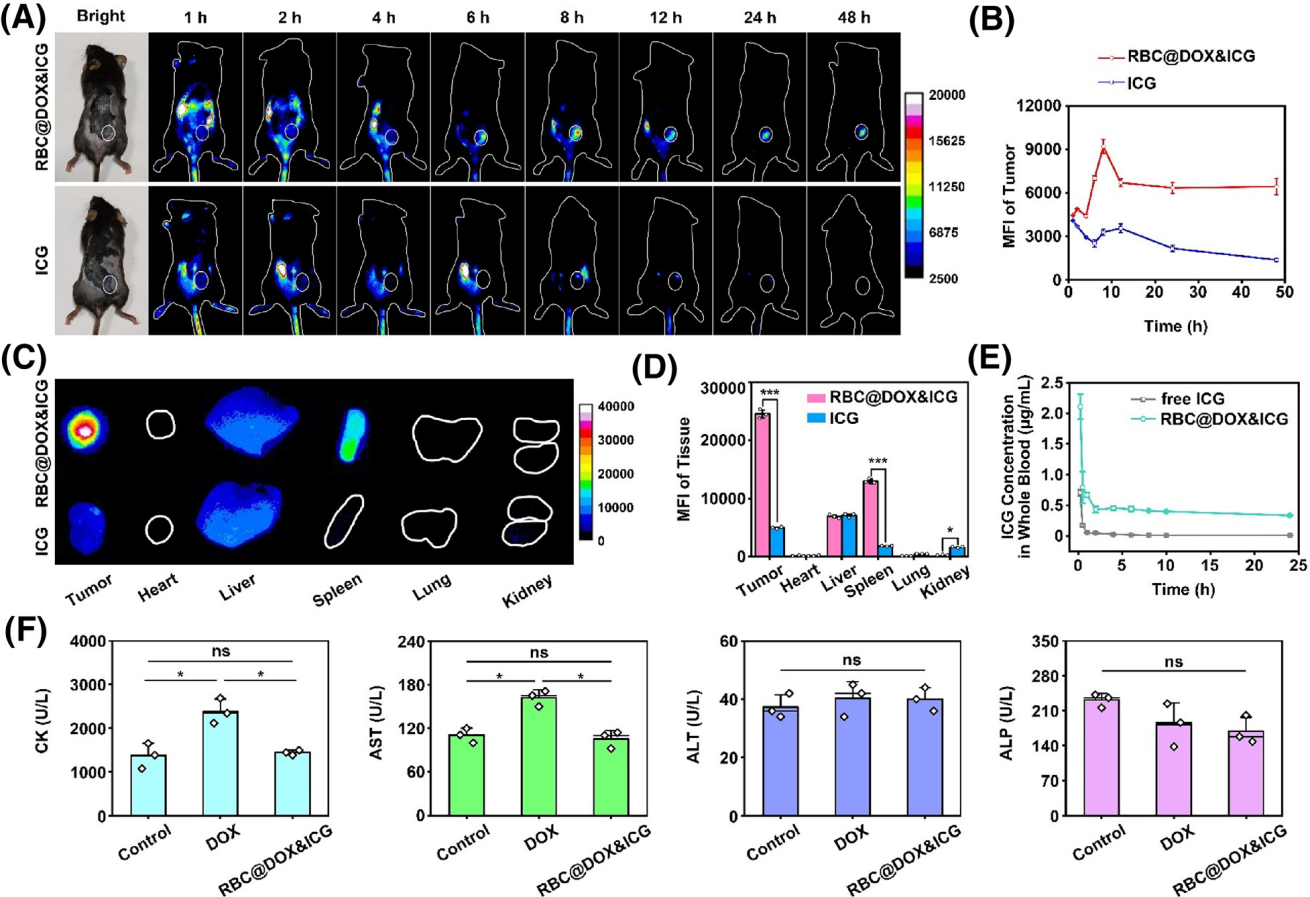
Biodistribution and biosafety of RBC@DOX&ICG *in vivo*. The tumor-bearing mice were used to assess the fluorescent navigation and blood circulation of RBC@DOX&ICG. The results showed that loaded RBCs improved tumor targeting and prolonged tumor retention of free ICG. (a) and (b) The whole-body NIR-II fluorescence imaging of tumor-bearing mice as a function of time after intravenous injection of RBC@DOX&ICG and free ICG (n = 3). (c) and (d) *Ex vivo* imaging of isolated tumors and major organs after 48 h of injection (^*^p < 0.05; n = 3). (e) ICG concentration in whole blood as a function of time after a single intravenous administration of RBC@DOX&ICG or the physical mixture of free ICG and DOX with a DOX-equivalent dose of 5 mg/kg (n = 3). (f) The blood biochemistry indexes of mice 2 days after *i.v.* injection of RBC@DOX&ICG and free DOX (ns, no significance; ^*^p < 0.05; n = 3).

After intravenous injection, we investigated the distribution of RBC@DOX&ICG in Hepa 1–6 tumor-bearing mice with a NIR-II fluorescence signal, while the mice injected with free ICG were used as control. As shown in Figs. 6(a) and S18, the fluorescence signal of free ICG is predominately located in the liver, which decreased quickly due to the clearance from the body. However, the fluorescence signal was much more obvious in the tumor of mice receiving RBC@DOX&ICG treatment, reaching a maximum after 8 h of injection [Fig. 6(b)]. After 48 h of injection, RBC@DOX&ICG was still retained at the tumor site. Afterward, the excised major organs were photographed to further confirm the biodistribution using the same NIR-II imaging system [Fig. 6(c)]. Compared with free ICG group, RBC@DOX&ICG displayed increased signals in the tumor and spleen. Semiquantitative analysis suggested that the MFI of tumor in the RBC@DOX&ICG group was about five times stronger than in the free ICG group [Fig. 6(d)]. In addition, we also detected the ICG and DOX content in major organs including tumor tissue to analyze bio-distribution of RBC@DOX&ICG, which compared with the physical mixture of free ICG and DOX at equivalent dose. As shown in Fig. S19, RBC@DOX&ICG showed much higher drug accumulation in tumor than physical mixture group in terms of both DOX and ICG after 8 h or 48 h of injection. For instance, at 8 h after injection, the tumor uptake in terms of DOX and ICG for the RBC@DOX&ICG group were 18.47 ± 1.61 and 12.84 ± 0.43%ID/g, respectively, as compared to 4.85 ± 0.20 and 3.33 ± 0.18%ID/g by the physical mixture (Fig. S19). In addition to tumor, other major organs also exhibited certain drug accumulations at both examined time-points. RBC@DOX&ICG demonstrated significantly decreased DOX accumulations within certain major organs like heart compared to the physical mixture, which might relieve its severe cardiotoxicity. The enhanced tumor-homing effect might be ascribed to the prolonged circulation of RBCs in favor of leaking out from blood vessel to permeate to the tumor sites through their membrane flexibility.^27^

To compare the *in vivo* pharmacokinetics of RBC@DOX&ICG to those of physical mixture, the whole blood at different time-points was collected to measure the drug concentrations using the spectrofluorometric assay. As displayed in Fig. 6(e), the ICG encapsulated in RBC@DOX&ICG displayed a much longer blood circulation than free ICG delivery as a formulation of physical mixture with free DOX. At 8 h post-injection, about 0.413 ± 0.008 *μ*g/ml of ICG was detected in the whole blood of mice administrated with RBC@DOX&ICG as compared to only 0.013 ± 0.003 *μ*g/ml of ICG detected in the physical mixture group. The pharmacokinetic profile originated from the DOX analysis presented a similar trend as ICG (Fig. S20), which also verified that RBC@DOX&ICG maintained a high concentration in the systemic circulation over a prolonged period. These results together suggested that RBCs as vehicles could help small molecules to escape phagocytosis and prolong blood circulation. In addition, we detected the blood biochemistry indexes to evaluate the *in vivo* acute toxicity of RBC@DOX&ICG or free DOX after 2 days. Untreated mice were used as the control group. All the parameters of RBC@DOX&ICG showed no significant difference in comparison with the control group, indicating their good safety *in vivo* after intravenous injection [Figs. 6(f) and S21]. However, as DOX has severe cardiotoxicity,^34,35^ the parameters reflecting cardiac muscle toxicity such as CK and AST in free DOX group were much higher than control and RBC@DOX&ICG groups, demonstrating that the RBC-based drug delivery system could alleviate the side effect of free DOX.

### F. Tumor microenvironment modulation by *μ*sPEF

**FIG. 7.**
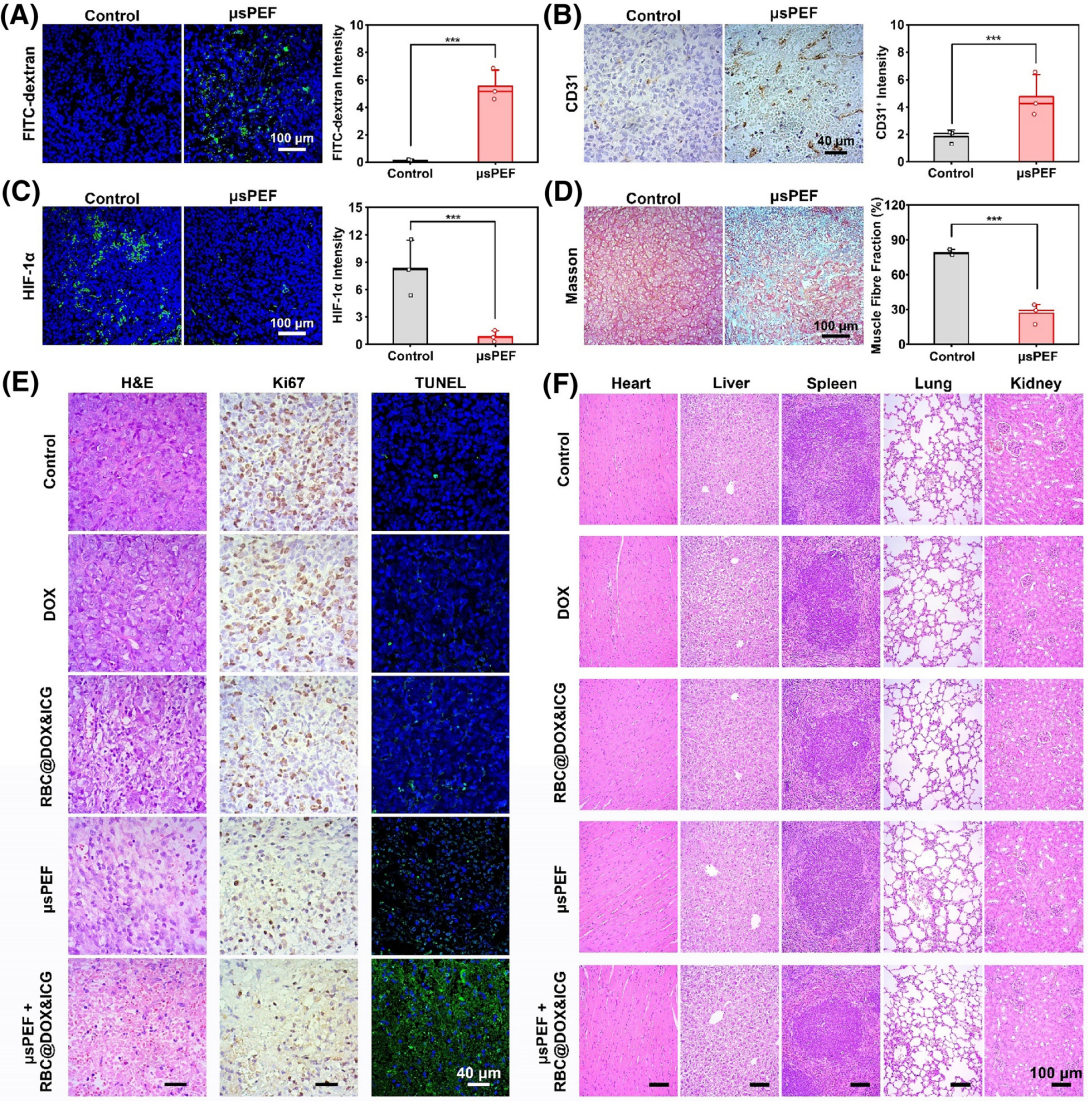
Impact of *μ*sPEF at 2500 V/cm on the tumor. By analyzing tumor sections, the effect of pulsed electric field on tumor microenvironment was studied. *μ*sPEF could increase the permeability and quantity of tumor blood vessels, relieve hypoxia, and soften dense stroma. In addition, *μ*sPEF + RBC@DOX&ICG represents great short-term therapeutic effects and no obvious signs of organ damage. (a) The fluorescence images of tumor sections of mice received *i.v.* injection of FITC-dextran. (b)–(d) Representative IHC staining of CD31, immunofluorescence staining of HIF-1α, and Masson of the tumor. (e) Optical microscopy images of tumor slices stained with H&E, antigen Ki67, and TUNEL after various treatments as indicated above. (f) The histopathological H&E staining of major organs after treatment. Three fields of view were randomly recorded and analyzed in (a)–(d) (^***^p < 0.001; n = 3).

Before the outset of *in vivo* antitumor therapy, we studied the capacity of *μ*sPEF to modulate the tumor microenvironment to potentiate the chemotherapy efficacy of DOX delivered by RBCs. First, to explore whether *μ*sPEF could increase the permeability of tumor vessels, FITC-labeled dextran (70 kD) was injected through *i.v.* injection after *μ*sPEF treatment. At 24 h post-injection, the tumor was collected for preparing frozen sections. The fluorescent intensity of FITC-dextran in the tumor section with *μ*sPEF treatment was significantly higher than that of the control group [Fig. 7(a)]. Immunohistochemical staining of CD31 was used to investigate the effect of *μ*sPEF on micro-vessel density [Fig. 7(b)]. The *μ*sPEF caused 2.6-times increase in microvessel density within the tumor site compared to the control group. In addition, immunofluorescent staining of hypoxia-inducible factor 1-alpha (HIF-1α) was used for determining the degree of hypoxia. Figure 7(c) showed that the mean expression of HIF-1α in control group was much higher than the *μ*sPEF group, with a 90% of decrease after *μ*sPEF treatment, verifying a great potential to relieve tumor hypoxia. The Masson staining showed that the tumor tissue became loose, as muscle fibers were dramatically degraded after *μ*sPEF treatment [Fig. 7(d)].^36^ Collectively, these results indicated that *μ*sPEF could reshape the tumor microenvironment by increasing the permeability and quantity of tumor blood vessels, relieving hypoxia, softening dense stroma, which would augment chemotherapy effect of DOX delivery by RBCs.

Furthermore, the H&E, Ki67, and TUNEL staining of tumor slices after 48 h of treatment were explored to observe the short-term therapeutic effect of different paradigms (control, DOX, RBC@DOX&ICG, *μ*sPEF, *μ*sPEF + RBC@DOX&ICG). As shown in Figs. 7(e) and S22, the H&E and Ki67 staining showed that RBC@DOX&ICG could lead to some cell necrosis and inhibit tumor cell proliferation, while free DOX group had little antitumor effect. Considering that RBCs can improve tumor targeting and prolong tumor retention (Figs. S18–S20), RBC@DOX&ICG exhibited a significant antitumor effect as compared to free DOX group owing to the increased tumor accumulation with the help of RBCs. In addition, *μ*sPEF itself also induced a large number of cell destruction due to its IRE ablation effect. Among all groups, the combination of *μ*sPEF and RBC@DOX&ICG induced most potent cancer cell apoptosis with much less proliferation and intact cells, proving the high efficacy of *μ*sPEF-controllable RBC@DOX&ICG. Meanwhile, the histopathological slices of the major organs were presented in Figs. 7(f) and S23, revealing no obvious signs of organ damage.

### G. *In vivo* antitumor effect

**FIG. 8.**
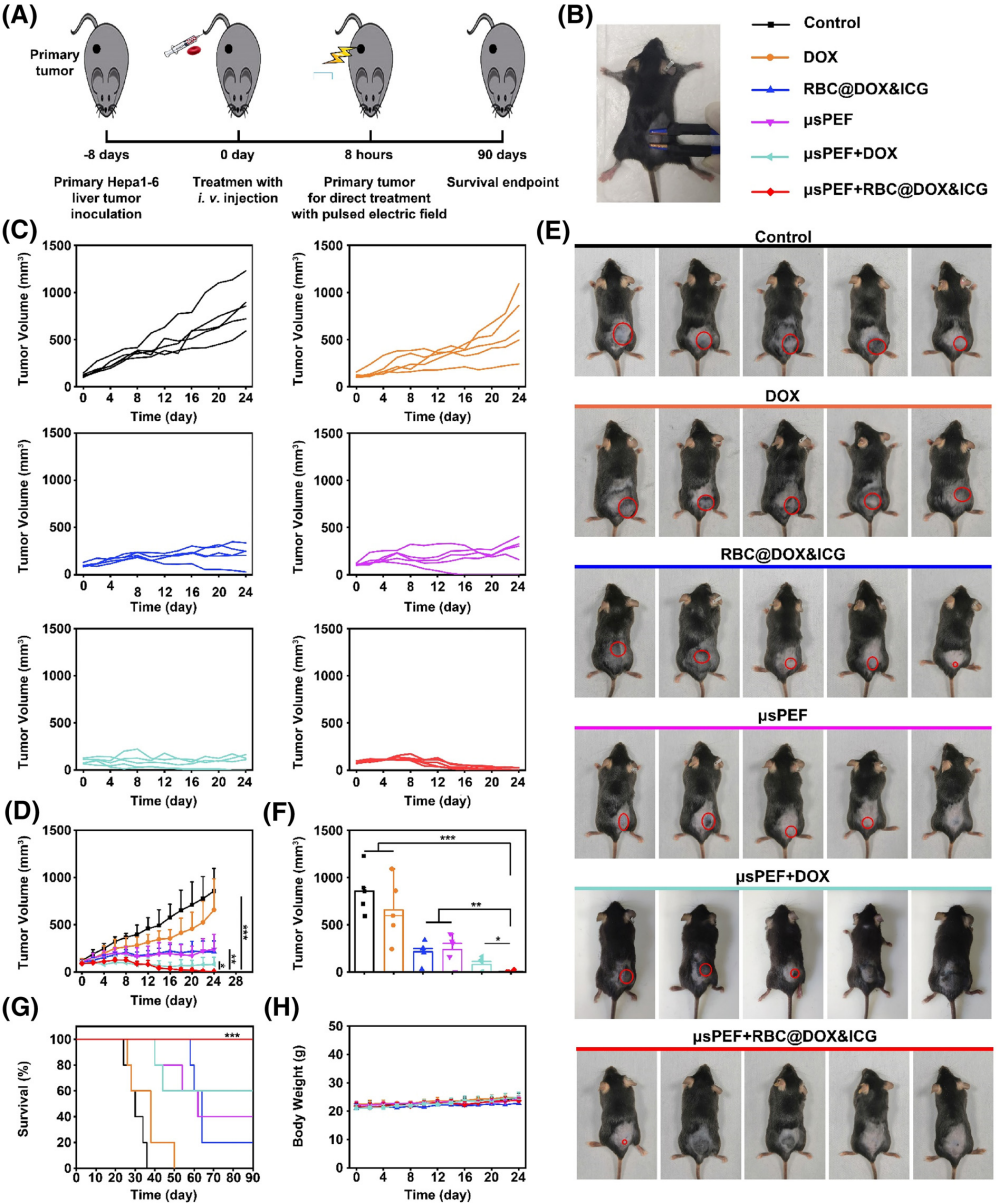
*In vivo* antitumor effect. Five groups of tumor-bearing mice were treated. After treatment, tumor volume was continuously monitored and photographed. The combination of *μ*sPEF at 2500 V/cm and RBC@DOX&ICG possesses an excellent antitumor effect. (a) Schematic view of the therapeutic procedure. (b) The photo of the tumor site implemented with the *μ*sPEF via the plate electrodes. (c) The *in vivo* tumor growth curve of different groups. (d) The tumor average growth curves of the mice received different treatments. (e) The photos of mice at day 24. (f) The average tumor volumes at day 24 (^*^p < 0.05, ^**^p < 0.01, ^***^p < 0.001; n = 5). (g) The survival curves of the mice in various groups (^***^p < 0.001; n = 5). (h) Body weight of the mice.

The *in vivo* antitumor effect of *μ*sPEF combining RBC@DOX&ICG was systemically investigated, according to the schedule outlined in Fig. 8(a). Hepa 1–6 tumor-bearing mice were randomly divided into five groups with five mice in each group. RBC@DOX&ICG was intravenously injected at day 0, and the *μ*sPEF-mediated tumor irradiation was performed after 8 h of injection. In the therapeutic course, the tumor volume and physical condition of mice were monitored continuously. Figure 8(b) was a photograph of the tumor site applied with the *μ*sPEF via plate electrodes. Detailed pulse parameters were as follows: electric field intensity: 2500 V/cm; pulse duration: 100 *μ*s; pulse repetition frequency: 1 Hz; number of repetition pulses: 40. As shown in Figs. 8(c) and 8(d), administration of free DOX once slightly inhibited tumor growth, while RBC@DOX&ICG transported more DOX to the tumor site and thus had a better tumor inhibition effect. Only *μ*sPEF could also restrain tumor growth, indicating its role of disturbing cell homeostasis to cause cell death. Most importantly, the combination of *μ*sPEF and RBC@DOX&ICG could quickly and completely remove cancer cells. On day 24, each mouse was photographed [Figs. 8(e) and S24], and their tumor volume was compared [Fig. 8(f)]. The average volume of control group was about 857 ± 239 mm^3^, while RBC@DOX&ICG or *μ*sPEF presented much less average volume around 200 mm^3^. Most obviously, the RBC@DOX&ICG and *μ*sPEF combination group exhibited the lowest average tumor volume of about 4.94 ± 11.05 mm^3^. The mice survival rate curves were shown in Fig. 8(g). Although single RBC@DOX&ICG or *μ*sPEF treatment remarkably restrained tumor growth, there was only a 20%–40% survival rate over 65 days. Encouragingly, the combination therapy significantly prolonged the survival rate up to 100% at the end of the trial period (day 90), with almost no tumor burden. Meanwhile, none of the treatments caused obvious body weight loss during the course of the study [Fig. 8(h)]. These results indicate that the combination of *μ*sPEF and RBC@DOX&ICG possesses an excellent antitumor effect, as a robust and well-tolerable technique for cancer therapy.

## III. DISCUSSION

In this word, based on the numerical simulation of RBC model under the pulsed electric field, the controlled drug release regulated by *μ*sPEF to induce the electroporation on the RBC membranes has been studied. *In vitro* experiments demonstrated that *μ*sPEF could effectively manipulate drug release and ablate tumor cells. Further *in vivo* experiments showed that RBCs were able to promote drug to accumulate at the tumor site and *μ*sPEF could modulate the tumor microenvironment to potentiate the chemotherapy efficacy. The antitumor experiment presented the excellent therapeutic effect with the combination of *μ*sPEF and RBC@DOX&ICG.

According to published reports,^37^ red blood cells are one of the most historical and popular natural drug delivery vehicles due to the excellent accessibility, great biocompatibility, tunable loading capacity, and long blood circulation half-life. Loading RBCs with DOX for tumor therapy can significantly prolong the drug's blood circulation, which helps the curative effect to last longer.^27,38^ However, these studies showed that the effect of drug-loaded RBCs alone was limited and cannot completely eliminate tumors. To improve their therapeutic effect, stimuli-responsive or controllable release behavior is a preferred choice. For instance, Sun *et al.* designed a NIR light stimulus-response RBC carrier to achieve a synergistic therapeutic effect through the combined photothermal-chemotherapy.^28^ However, probably due to the limited light penetration, the study only investigated the *in vitro* performance. In addition, our group previously fabricated a RBC delivery system for NIR II fluorescence bioimaging-guided tumor surgery and light-triggered O_2_ release to enhance photodynamic therapy, but could only achieve a short-term effect with rapid tumor recurrence.^29^ With regard to electric field as an external stimulus without the limitation of tissue penetration, Zhao *et al.* employed an implantable magnet triboelectric nanogenerator (MTENG) to *in situ* control the drug release from the DOX loaded RBCs.^22^ However, the electric field collected from self-powered triboelectric nanogenerator was not strong enough and could not be adjusted artificially, and the therapeutic effect was still suboptimal as the survival time of a large portion of mice was found to be less than 60 days. Compared with triboelectric field with low intensity, irreversible electroporation approved by FDA and NMPA in clinic can directly kill cancer cells and control drug release, which lead to a high therapeutic efficiency with the survival time of all treated mice increasing above 90 days without tumor burden.

Looking into the future, more critical issues need to be addressed. From the perspective of reproducibility, easy for manipulation (both time and cost), and palpable detection of tumor growth, we established a subcutaneous tumor, which is also the most common model reflecting the regular growth of human tumors to investigate the *in vivo* performance of RBC@DOX&ICG incorporated with *μ*sPEF. However, this model fails to fully mimic a clinical situation. For example, tumor heterogeneity is difficult to take into account, as in most scenarios, homogeneous cell lines are used to construct a tumor model. In addition, subcutaneous tumors do not grow in their native tumor microenvironment and thus lose tumor–stromal interactions. With this regard, patient-derived xenografts (PDX) implanted *in situ* or spontaneous tumor model will be more meaningful, which will be considered in our future work. In addition, to prove the feasibility of this treatment in humans in future, human RBCs were used to be stimulated by *μ*sPEF. In response to the electric field, the release behavior of hemoglobin from human RBCs is similar with murine RBCs (Fig. S25), but how to realize precise drug release *in vivo* is a huge task, considering the complicated environment of tumor tissues and non-uniform distribution of electric field in them. In the setting of antitumor treatment, the therapeutic paradigm that can maximize drug release/bioavailability to induce cancer damage is more anticipated. Based on these, we selected the 2500 V/cm as the parameter to complete destruction of the RBC to trigger maximal DOX release in the anticancer experiments. However, for some other specific diseases such as diabetes, cardiovascular disorders, and neurodegeneration, precise control of dosage is very important for the success of any drug-based therapy. This work only provides a proof-of-concept of *μ*sPEF to realize precise drug release, and there is still a huge gap to bridge this strategy to the clinical requirements.

## IV. CONCLUSION

In summary, we presented a controlled dosage regimen for cancer treatment by the combination of IRE (*μ*sPEF) and a cell-based drug delivery system (RBC@DOX&ICG). The *μ*sPEF output by our homemade high-voltage pulse generator could quantitatively control drug release from RBC as the DDS model, which was verified by mathematical simulation and actual measurement. Furthermore, the NIR-II fluorescence of ICG in RBC preferentially accumulated at the tumor site after intravenous injection, which allowed the guidance of electrodes arrangement for the IRE-controllable RBC carrier in tumor-bearing mice and displayed an excellent tumor growth inhibition with the survival rate up to 100% at the end of the trial period (day 90), through IRE-mediated tumor ablation, drug release, and tumor stroma modulation. Therefore, this presented work provided a novel tumor therapeutic paradigm using IRE-controllable cell-based DDS, which would be highly promising for clinic translation.

## V. METHODS

### A. 3D finite-element simulation of the RBC

Multiphysics COMSOL finite-element analysis software is used to establish a 3D RBC model whose electrical characteristics are defined by conductivity and permittivity, with every part of the RBC deemed as isotropic, linear, and homogeneous media. The size of the RBC model was built by averaging the size of dozens of RBCs. The free tetrahedral mesh is used to form a complete mesh. As shown in Fig. S1, *R_c_*, *r_c_*, *T_c_*, and *t_c_* are the dimension parameters of the red blood cell in the simulation. *λ_m_*, *λ_mem_*, and *λ_c_* are the media conductivity, membrane conductivity, and cytoplasm conductivity, respectively. *ε_m_*, *ε_mem_*, and *ε_c_* are the media permittivity, membrane permittivity, and cytoplasm permittivity, respectively. All parameters are described in Fig. S2. One square wave pulse with different field strength was used. The detailed mathematical formulas are presented in the supplementary material.

### B. Preparation of RBC@DOX&ICG

Whole blood was obtained from the orbital sinus of C57BL/6 mice. The RBCs were separated from whole blood by centrifugation (4 °C, 800 g, 5 min) and then washed three times with pre-cooled PBS before re-suspending it in pre-cooled PBS (50% hematocrit). The DOX and ICG-BSA were loaded into the RBCs by the “hypotonic dialysis.” 1 ml mixture containing 500 ug DOX and 60 mg ICG-BSA complex (with 1 mg of ICG) was added to 1 ml suspended RBCs. Then all mixture was transferred into a dialysis bag with a 3500 Da molecular weight cutoff. The hypotonic dialysis was carried out at 4 °C lasting 30 min in the buffer (pH 7.4), containing 10 mM Na_2_HPO_4_, 10 mM NaH_2_PO_4_, 20 mM Glucose, 4 mM MgCl_2_, 2 mM ATP, and 3 mM reduced glutathione. The resealing process was obtained by dialyzing RBCs in a hypertonic solution (pH 7.4) including 10 mM sodium pyruvate, 10 mM inosine, 10 mM glucose, 3.5 mM NaH_2_PO_4_, 0.5 mM adenine, 2 mM ATP, 3 mM reduced glutathione, and 1.2% (w/v) NaCl at 37 °C for 30 min. The RBCs were centrifugated (4 °C, 800 g, 5 min) and washed three times with pre-cooled PBS to remove redundant drugs.

### C. Characterization of RBC@DOX&ICG

The fluorescence images of drug-loaded RBCs were obtained by a Zeiss LSM780 laser scanning confocal microscope. The magnification of the microscope was set at 40×. For the excitation and emission wavelengths of the fluorescent dyes, DOX was excited at 514 nm, and its emission was recorded at 583 nm. On the other hand, ICG was excited at 633 nm, and its emission was recorded at 693 nm. DOX loading in RBCs was measured by a flow cytometer (BD, FACSAria TM III, USA). The fluorescence spectrum of DOX was used to determine DOX loading. To quantitate the DOX loading, the RBC membranes were destructed completely using a lysis buffer, while the released DOX was extracted with an acetonitrile solvent and measured by high-performance liquid chromatography. The amount of ICG–BSA loading was measured according to the ICG-BSA absorbance at 790 nm. NIR-II fluorescence image was collected to verify ICG-BSA loading inside RBCs. Carrier-RBC membrane damage (PS exposure) was quantified using FITC-Annexin V assay (Dojindo) and analyzed by flow cytometry.

### D. Equipment of pulse transmitter

The high-voltage pulse generator used in the experiment was homemade in the laboratory. It was developed using the Marx circuit enabled delivering microsecond pulses with a maximum voltage of 5 kV, the shortest length of 10 *μ*s, and a repetition frequency from 1 Hz to 1 kHz. The output voltage and current were measured by a HDO6054 500 MHz high-definition oscilloscope (Teledyne LeCroy Inc., USA) with a PEF-5 kV high-voltage probe (Teledyne LeCroy Inc., USA) and a Pearson current probe 411 (Pearson Electronics Inc., USA). The waveform of microsecond pulse with 100 *μ*s duration and various magnitude is shown in Fig. S3.

### E. Electric release experiments

The suspensions of 10% hematocrit RBC@DOX&ICG were prepared and then 100 *μ*l aliquots of the suspension were stimulated in a 2 mm-gap cuvette (Harvard Apparatus, USA) embedded with two aluminum plate electrodes. Pulse parameters are as follows: electric field intensity: 500–3000 V/cm; pulse duration: 100 *μ*s; pulse repetition frequency: 1 Hz; the number of repetition pulses: 40. After treatment, the samples were subjected to centrifugation immediately. The supernatant was collected for recording the absorption spectrum at the wavelength of 350–600 nm and the fluorescence spectrum at 560–650 nm. The suspensions in the cuvette before and after treatment were photographed using the IR thermal camera (Ti25 Fluke Co, USA). For scanning electron microscopy characterization, the sediments were collected by centrifugation (800 g, 5 min) and incubated in electron microscopy stationary liquid (2.5% glutaraldehyde, Servicebio). A detailed sample preparation was performed following the reported recommendations.^23^ For *in vivo* drug release, RBC@DOX&ICG (200 *μ*l, 50 *μ*g/ml of ICG) was injected into the tumor-bearing mice *i.v.* and *μ*sPEF was applied after 8 h. Then, the tumors were collected to acquire the freezing microtome section, which were dyed with DAPI and visualized using the confocal fluorescence microscope.

### F. *In vitro* anti-cancer effect

Hepa 1–6 cells were cultured in Dulbecco's modified Eagle's medium (DMEM, Gibco) containing 10% fetal bovine serum (FBS, Gibco) and 1% penicillin/streptomycin (Gibco) in a 37 °C humidified incubator with 5% CO_2_. The cells were collected and seeded onto the 24-well plates at a concentration of 10^5^ cells/ml. Over 1 day, before the treatment, the medium was removed and replaced with 0.5 ml of fresh medium with RBCs, RBC@DOX&ICG, or free DOX (5 *μ*g/ml). The electric pulses were delivered through two 1 mm outer diameter stainless steel needle electrodes with a 2 mm edge-to-edge distance between the electrodes. The parameters of *μ*sPEF were as follows: electric field intensity: 2500 V/cm; pulse duration: 100 *μ*s; pulse repetition frequency: 1 Hz; the number of repetition pulses: 40. After 2 h, the RBCs were swept by PBS and replaced with fresh medium. After 24 h of incubation, live cells were stained with calcein-AM to display green fluorescence. The ablation areas were visualized using a fluorescence microscope (Zeiss Axio Vert. A1, Germany). To delineate the boundary between the green and black maps representing the ablation area, ImageJ software was utilized. The contrast of the images was adjusted to ensure consistency across the dataset. Subsequently, the images were converted to an 8-bit binary format. Stacks of binary images were then processed using the Analyze Particle function, which facilitated the identification and quantification of the ablation regions' areas. Additionally, any holes present within the outlined regions were automatically filled by the Analyze Particle function.

### G. *In vivo* distribution

Male C57BL/6 mice aged 4–5 weeks and weighing 15–20 g were obtained from Wushi, Inc. Corp. Subcutaneous tumor models were established with an injection of 3 × 10^6^ Hepa 1–6 cells per mouse. While the tumor volume is about 100 mm^3^, ICG (200 *μ*l, 50 *μ*g/ml) or RBC@DOX&ICG (200 *μ*l, 50 *μ*g/ml of ICG) was intravenously injected into the tumor-bearing mice. At 1, 2, 4, 6, 8, 12, 24, and 48 h after injection, a NIR-II imaging system was used to observe the fluorescent signals. Afterward, the mice were sacrificed. The heart, liver, spleen, lung, kidney, and tumor were collected and imaged. In addition, we also detected the drug content in these tissues using spectrofluorometric assay. The major tissues were weighed and then cut into small pieces and homogenized with 400 *μ*l 1% Triton-100, and DOX/ICG was extracted with 800 *μ*l acetonitrile. After incubation on ice for 15 min, the mixture was centrifuged at 13 000 rpm for 10 min, and the supernatant was collected to determine the DOX (Ex/Em, 480/590) and ICG (Ex/Em, 780/820) concentration via a steady state and time-resolved photoluminescence spectrometer (Edinburg, FLS1000, EI).

### H. The pharmacokinetics of RBC@DOX&ICG

The healthy mice were *i.v.* injected with RBC@DOX&ICG or the physical mixture of ICG and DOX at an equivalent dose of 100 *μ*g DOX and 25 *μ*g ICG per mouse. At different time points (1, 2, 4, 6, 8, 10, and 24 h), 50 *μ*l of blood was collected from the tail vein into heparinized tubes with 200 *μ*l double distilled water. Then, the blood was mixed with 750 *μ*l acetonitrile to precipitate all the proteins, left on ice for 15 min, and centrifuged at 13 000 rpm and 4 °C for 10 min. After centrifugation, the supernatant was collected and detected by a steady state and time-resolved photoluminescence spectrometer (Edinburg, FLS1000, EI) to determine the DOX (Ex/Em, 480/590) and ICG (Ex/Em, 780/820) levels.

### I. The biosafety evaluation *in vivo*

For the biosafety evaluation of therapy, the treated mice were sacrificed in 2 days, and the visceral organs (heart, liver, spleen, lung, and kidney) were isolated for H&E examination. In addition, the serum biochemistry analyses were carried out to assess the toxicity of DOX (500 *μ*l, 200 *μ*g/ml) and RBC@DOX&ICG (500 *μ*l, 200 *μ*g/ml of DOX). After 2 days of treatment, the serum was isolated from blood sampled by eyeball extirpating and analyzed using an automated hematology analyzer.

### J. Tumor microenvironment modulation by *μ*sPEF

The tumor-bearing mice were treated with 40 *μ*s pulses (electric field intensity: 2500 V/cm; pulse duration: 100 *μ*s; pulse repetition frequency: 1 Hz). After 2 days, the animals were euthanized and the tumors were collected to fix, embed, and slice. The Masson trichrome stain was used to analyze the change of tumor density by *μ*sPEF. The immunohistochemical staining of CD31 was carried out to assess tumor microvessel density. The hypoxia-inducible factor 1-alpha (HIF-1α) expression after IRE was also observed. The immunohistochemical slices were image by optical microscope. To determine whether *μ*sPEF increased the permeability of tumor blood vessels, the treated mice were *i.v.* injected FITC-conjugated dextran (70 kD). After 24 h, the tumors were collected to acquire the freezing microtome section. Frozen sections were dyed with DAPI and visualized using the confocal fluorescence microscope.

### K. *In vivo* antitumor effect

To evaluate the treatment effect, 30 mice were divided into five groups (n = 6, each group) with Hepa 1–6 tumor whose volume reached about 100 mm^3^. The details were as follows: (1) PBS; (2) DOX (500 *μ*l, 200 *μ*g/ml); (3) RBC@DOX&ICG (500 *μ*l, 200 *μ*g/ml of DOX); (4) *μ*sPEF; (5) *μ*sPEF and RBC@DOX&ICG (500 *μ*l, 200 *μ*g/ml of DOX). The parameters of *μ*sPEF were as follows: electric field intensity: 2500 V/cm; pulse duration: 100 *μ*s; pulse repetition frequency: 1 Hz; the number of repetition pulses: 40. The pulses were implemented using plate electrodes 8 h after loaded-RBCs were injected into the tail vein. The antitumor effect was observed by measuring the body weights and the tumor volumes every 2 days. The tumor volume was calculated by the following: tumor volume = length × width^2^/2. In order to observe the histopathological changes of the tumor, 2 days after treatment, one tumor-bearing mouse in each group was killed. The tumor was removed, fixed by formalin solution, embedded in paraffin, and sliced to stain. H&E, Ki67, and TUNEL were analyzed to evaluate the histological damages.

### L. Statistical analysis

All experimental results are expressed as the mean ± SD. The data processing was carried out by Origin 2018. The one-way analysis of variance (ANOVA) method was used to judge statistical significance. Survival time was assessed by the Kaplan–Meier method, and statistical differences were judged with a log-rank test. ^*^p < 0.05, ^**^p < 0.01, and ^***^p < 0.001. The P-value < 0.05 was considered as statistical significance.

## SUPPLEMENTARY MATERIAL

See the supplementary material for the additional results of schematic diagram of a RBC model, parameters of the electroporation model, the voltage and current waveform of pulse, the high magnification images of RBCs, the calibration curve of DOX and ICG, the absorption spectra and fluorescent spectra of ICG and ICG-BSA, FITC-Annexin V analysis, fluorescence signal of the blood collected from mice after tail vein injection of the DiI labeled native RBCs or treated RBCs, the absorption spectra of native RBCs or treated RBCs, sensitivity of loaded RBCs at 1.0% hematocrit to different stress conditions, the photograph of pulsed electric field equipment, the image of RBC@DOX&ICG taken immediately after *μ*sPEF treatment, the temperature change of the RBC@DOX&ICG suspensions in the cuvette, florescence image of tumor cells in the different groups and the mean fluorescence intensity, the original high-resolution unedited images of the whole mice and organs, bio-distribution profiles of total DOX and total ICG at different times, DOX concentration in whole blood, the blood biochemistry indexes of the mice, the absorption spectrum of murine and human RBCs' supernatant after treatment, and the methods of 3D finite-element simulation.

## Data Availability

The data that support the findings of this study are available within the article and its supplementary material.
